# Toothbrush deterioration and parents’ suggestions to improve the design of toothbrushes used by children with special care needs

**DOI:** 10.1186/s12887-020-02347-8

**Published:** 2020-09-21

**Authors:** Ni Zhou, Hai Ming Wong, Colman McGrath

**Affiliations:** 1 Department of Paediatric & Preventive Dentistry, Affiliated Stomatology Hospital of Kunming Medical University, Kunming, China; 2 Paediatric Dentistry & Orthodontics, Faculty of Dentistry, The University of Hong Kong, 2/F Prince Philip Dental Hospital, 34 Hospital Road, Sai Ying Pun, Hong Kong SAR, China; 3 Periodontology and Public Health, Faculty of Dentistry, The University of Hong Kong, 2/F Prince Philip Dental Hospital, 34 Hospital Road, Hong Kong SAR, China

**Keywords:** Toothbrush, Wear, Bite marks, Pediatric, Special needs, Design

## Abstract

**Background:**

Biting objects was a parafunctional oral habit among children with special care needs. Chewing or biting toothbrushes could expedite the process of toothbrush wear. However, few studies evaluated the deterioration levels of toothbrushes used by children with special needs. This study aimed to assess the deterioration level of toothbrushes used by children with special care needs, and collect parents’ feedbacks to improve the design of children’s toothbrushes.

**Methods:**

The cross-sectional study recruited 277 children who had special care needs. Children’s toothbrushing behaviors, background information, and parents’ comments on toothbrushes were obtained. Toothbrush deterioration was assessed by bristle wear and bite mark scores. Higher scores indicated severe deterioration.

**Results:**

Three hundred twenty-one toothbrushes were collected. Children who used 2 to 6 toothbrushes in a 3-month period showed higher toothbrush deterioration scores than children who used a single toothbrush. Over 40% children’s toothbrushes presented excessive wear. Excessive wear was associated with social skills and parents’ education background. Distinct bite marks tended to exist on toothbrushes which had been used by children who showed challenging behaviors during toothbrushing (OR = 1.96, 95%CI1.15–3.32, *p* < 0.05). Approximately 27% parents reported that children’s toothbrushes should be modified. Parents recommended that the size of toothbrush heads, the angle of handles, and the texture/length/distribution/diameter of bristles should be adjusted. Besides, ideal toothbrushes should be able to provide verbal or visual instructions to children, motivate children to brush teeth, simplify toothbrushing procedure, and protect children who had toothbrush-biting habits.

**Conclusions:**

Excessive wear and distinct bite marks can be found on toothbrushes that had been used by children with special care needs. Toothbrush deterioration was associated with children’s social skills, toothbrushing behaviors, and parents’ educational attainment. The commercially available toothbrushes should be modified to meet the additional needs of young children.

## Background

Toothbrushes are the primary tools for dental plaque control and dental disease prevention [1, 2]. If misused, toothbrushes can have adverse effects on soft tissues and hard dental tissues. It was documented that gingival abrasion, abrasion of dentin, bristle impaction, or ulceration could be caused by traumatic toothbrushing [1, 3, 4]. Various factors can contribute to the traumatic toothbrushing, including, brushing force, brushing technique, type of toothbrushes, component of toothpaste, texture or shapes of toothbrush bristles, and the time of use [4–7]. Although toothbrushes were selected properly and appropriate brushing technique was used, the morphological features of toothbrushes could change with time. Most dentists recommended that toothbrushes should be renewed every 2 to 3 months [8]. Since the tufts of worn brushes could compromise their abilities to remove food debris and dental plaque, the splaying of toothbrush bristles could be considered as an indicator for toothbrush replacement [9].

Children’s toothbrushes require additional attention and monitoring, especially toothbrushes used by younger children [5, 10, 11]. It was reported that the tips of toothbrushes used by preschool children were likely to be cracked after 4 weeks, and the cracked tips could serve as a habitat for microorganisms [5]. The bristles of toothbrushes used by children are vulnerable to be matted, as they brush their teeth with uneven strokes, and chew or bite the toothbrushes during brushing [11]. Biting objects was reported to be a common parafunctional oral habit among children with special care needs, and biting toothbrushes could expedite the process of toothbrush wear [9, 12].

Numbers of studies have evaluated the severity of toothbrush wear [9, 13, 14]. However, few studies investigated the distribution of bite marks on toothbrushes that had been used by children with special care needs. In this study, toothbrushes used by preschool children with special care needs were collected, aiming to evaluate the deterioration levels of toothbrushes used by young children with special care needs and collect parents’ feedbacks to improve the design of toothbrushes for those children.

## Methods

### Study design and participant recruitment

This observational study was conducted in the Special Child Care Centers (SCCCs). Ethical approval was granted by the local Institutional Review Board of Ethics (file No: UW 16–012). The study was in accordance with the guidelines of the Strengthening the Reporting of Observational Studies in Epidemiology (STROBE) as detailed in online supplementary material. During June 2016 to January 2017, invitation letters, consent forms, and self-seal plastic bags were delivered to 16 SCCCs. Each plastic bag was attached with a label, showing the instructions for toothbrush collection. The recruited children were aged between 2-to-6 years, and they were diagnosed with disabilities. Their parents were invited to collect the toothbrushes which had been used by their children during the past 3 months and seal the used toothbrushes in the plastic bags. Children whose parents did not sign the consent forms were excluded.

### Outcome measurement

**Fig. 1:**
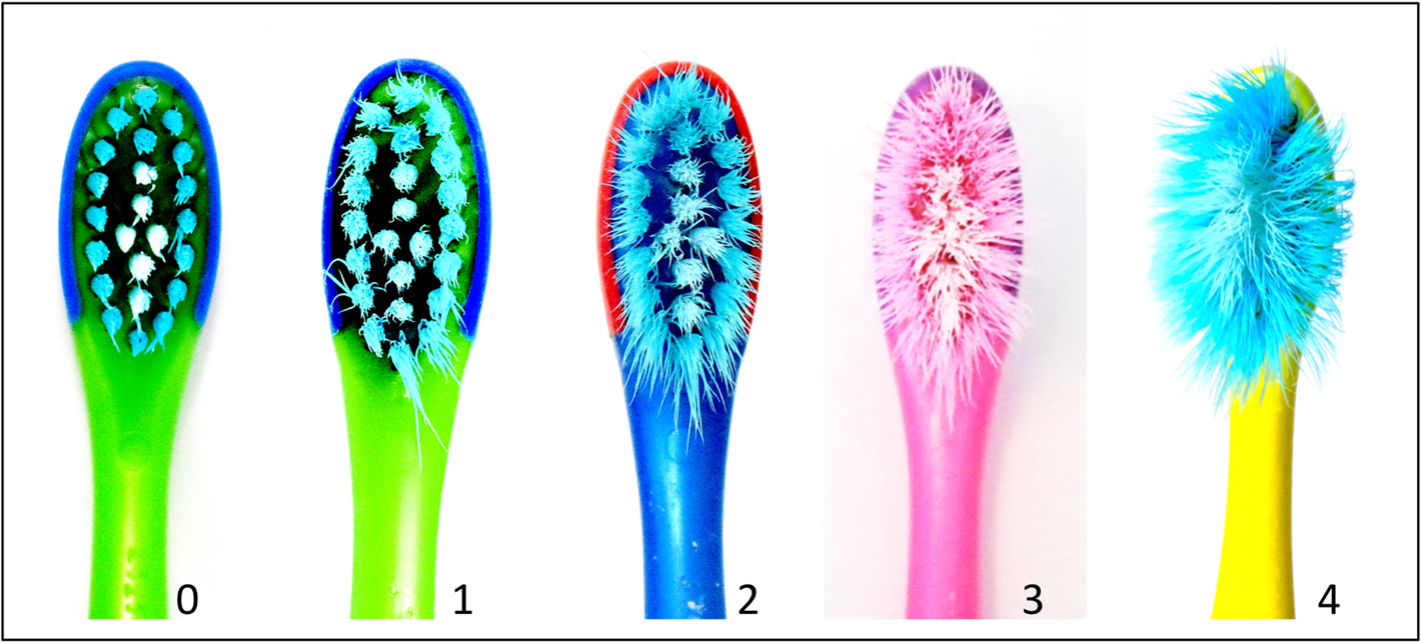
Brush wear scores proposed by Conforti et al. [9, 14] The above images were not original pictures from the references

The severity of the toothbrush deterioration was assessed by toothbrush wear and the distribution of bite marks on the collected toothbrushes (Table 1). Toothbrush wear was rated by a 5-point scale (Fig. 1), ranging from 0 (no visible signs of bristle wear) to 4 (extreme signs of bristle wear) [9, 14]. Since the splaying of the outer tufts beyond the base of the toothbrush (presence of medium wear) was a condition that indicated toothbrush replacement [9], the coding of 3 to 4 was considered as excessive wear. A single trained and calibrated investigator assessed the degree of toothbrush wear. The kappa value was 0.84 for re-examination of 10% collected toothbrushes, indicating good intra-rater reliability.

**Table 1:** Coding of toothbrush wear and bite marks on the toothbrushes

Coding	Degree	Toothbrush wear (Conforti et al.,2003 [14])	Distribution of bite marks
0	No	No visible signs of wear	Absence of bite marks
1	Light	Inner tufts are intact; outer tufts begin to splay	Presence of blurred marks
2	Medium	Inner tufts begin to splay; outer tufts splayed beyond the base of the toothbrush	Localized distinct marks
3	Heavy	Inner and outer tufts are splayed	Generalized distinct marks
4	Extreme	All the tufts are splayed whereby no distinction can be made	Distinct marks distributed on the entire surface; the contour of the toothbrush head is significantly distorted

**Fig. 2:**
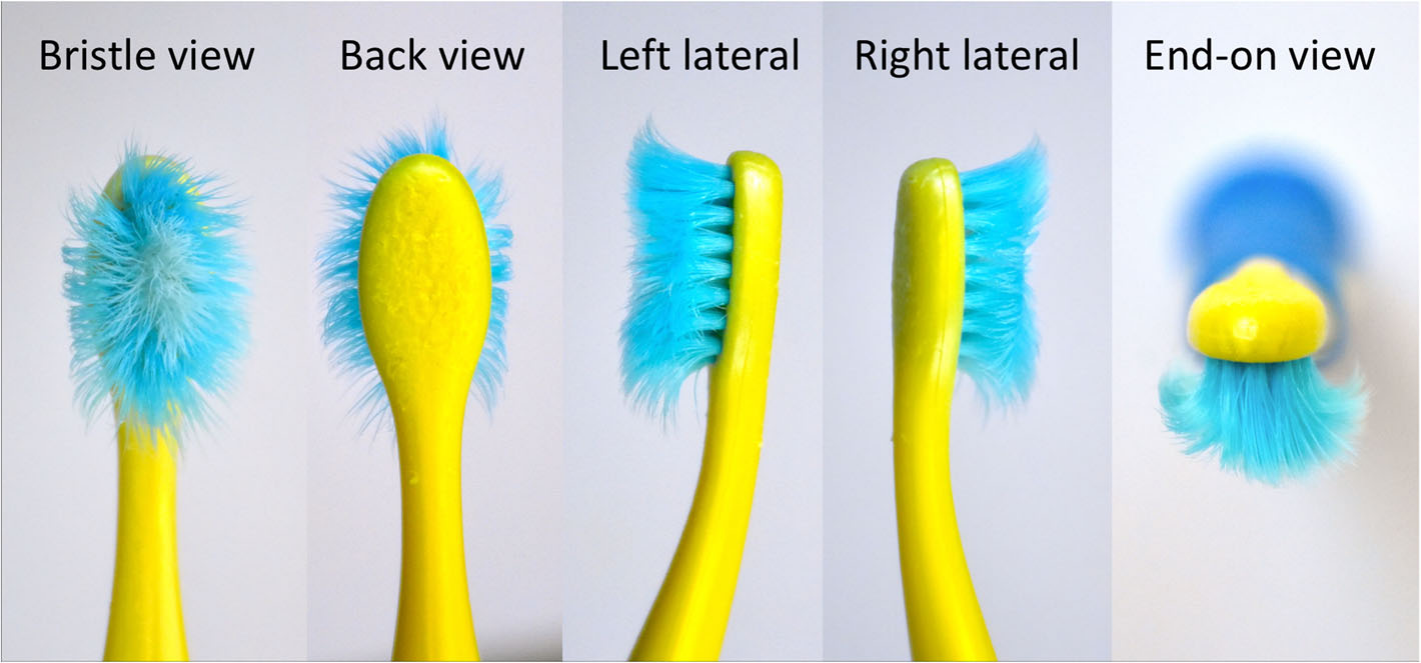
Photographs taken from five views

**Fig. 3:**
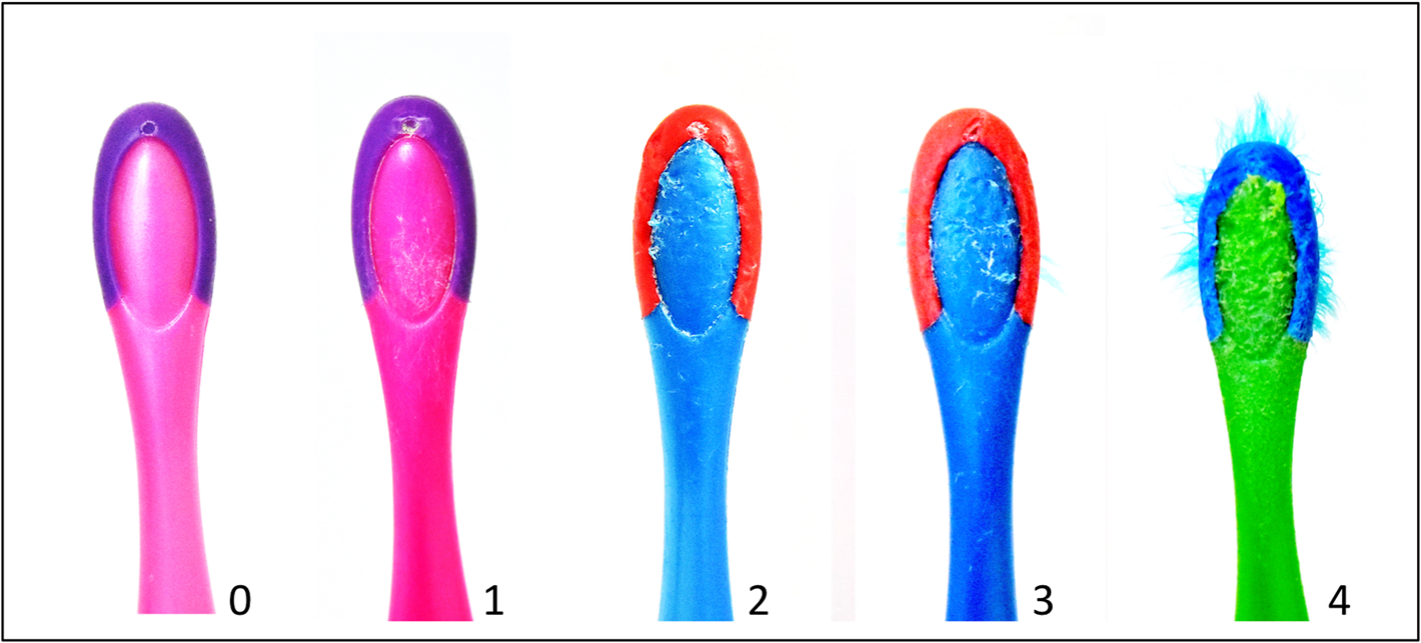
Bite mark scores on the toothbrushes used by children with special care needs

The distribution of bite marks on the collected toothbrushes was rated by a 5-point scale (Table 1). The scale had been validated in the pilot study, in which a convenience sample of 150 toothbrushes was collected. A series of five photographs were taken for each toothbrush, including a bristle view, posterior view, end-on view, left and right lateral view (Fig. 2). As bite marks were most likely to be observed on the back-view images, it was selected to be the index view for bite mark assessment. The rating criteria were demonstrated by a description (Table 1), along with a back-view image of the morphological features (Fig. 3). Two investigators rated the bite-mark scores, following the above illustrations. The coding 2 to 5 indicated that there were distinct bite marks on the toothbrush heads. By using the rating scale, the toothbrush deterioration caused by biting or chewing was positively correlated to the degree of toothbrush wear (r = 0.64, *p* < 0.01). The Kappa value was 0.73, indicating that the inter-examiner reliability was substantial. The Cronbach’s Alpha value was 0.97, suggesting that the internal consistency of the rating scale was excellent.

Children’s demographic information, primary diagnosis, adaptive functioning skills were collected [15]. The questionnaires regarding children’s toothbrushing behaviors (“Is toothbrushing a difficult task for your child: Yes/No”; “Which type of toothbrush has been used by your child: Manual toothbrush/ Electric toothbrush/ both manual and electric toothbrushes”; “How often does your child brush his/her teeth: Never/occasionally/Once daily/Twice daily or more”; and “How long does your child brush his/her teeth each time: Less than 1 minute/ 1-2 minutes/ 2-3 minutes/ Over 3 minutes”) and parents’ comments about the children’s toothbrushes (“Do you think the toothbrush for your child need to be modified: No/Yes, please list your suggestions”) were filled by parents.

### Statistical analysis

Statistical analysis was performed by IBM SPSS Statistics (Version 26.0. Armonk, New York: IBM Corp). Data analysis was performed at the toothbrush level and child level. When analyzed at the child level, if a child had used two or more toothbrushes during the past 3 months, all the toothbrushes were rated. The highest score was regarded as the final score for the child. Correlations between wear scores and bite-mark scores were estimated by the Spearman Correlation Coefficients. Binary Logistic Regression was performed to identify the factors which might be related to the presence of excessive toothbrush deterioration. Children’s primary diagnosis, adaptive skills, toothbrushing behaviors, parents’ educational attainment, and household income were entered into the full models. By using the backward method, odds ratio (OR) and 95% confidence interval (CI) were presented in the final models. The significance level was set at 0.05.

## Results

### Participant characteristics

A total of 277 children aged between 2 to 6 years were recruited. A quarter of them were diagnosed with developmental delays, 86 (31.0%) were on autism, and the remaining had epilepsy, cerebral palsy, Down syndrome, or multiple developmental disorders. 60.6% children came from high-income families, 58.5% parents received at least 14 years’ education, and 61.7% parents reported that toothbrushing was a difficult task for their children. Most (93.1%) children used manual toothbrushes, and 12 (4.3%) children spent more than 3 min in toothbrushing (Table 2).

**Table 2:** Participant characteristics

Characteristics	N (%)
Age (yr) ± SD	3.64 ± 0.78
Male	187 (67.5)
Diagnosis	
Autism	86 (31.0)
Developmental delay	71 (25.6)
Down syndrome (DS), Cerebral Palsy (CP), or epilepsy	32 (11.6)
Multiple diagnoses	88 (31.8)
Conceptual skills	
Average or high	38 (13.7)
Limited	171 (61.7)
Low	68 (24.5)
Practical skills	
Average or high	24 (8.7)
Limited	142 (51.3)
Low	111 (40.1)
Social skills	
Average or high	43 (15.5)
Limited	175 (63.2)
Low	59 (21.3)
Parents’ educational attainment	
9 years or below	37 (13.4)
10–13 years	78 (28.2)
14 years or above	162 (58.5)
Household monthly income (HKD)	
20,000 or below	109 (39.4)
Above 20,000	168 (60.6)
Toothbrushing is a difficult task	171 (61.7)
Types of toothbrush	
Manual	258 (93.1)
Electric	12 (4.3)
Both manual and electric	7 (2.5)
Toothbrushing frequency	
Never/occasionally	44 (15.9)
Once daily	89 (32.1)
Twice daily or more	144 (52.0)
Toothbrushing duration	
Less than 1 min	76 (27.4)
1–2 min	142 (51.3)
2–3 min	47 (17.0)
Over 3 min	12 (4.3)

### Characteristics of children who used multiple toothbrushes in a 3-month period

Most (91.0%) children used a single toothbrush in 3 months, while the others used 2 to 6 (multiple) toothbrushes during the past 3 months (Table 3). Children used multiple toothbrushes in a 3-month period showed significantly higher wear scores (3.24 ± 0.83vs. 1.75 ± 1.43, *p* < 0.001) and bite scores (2.76 ± 1.36vs.1.28 ± 1.34, *p* < 0.001) than those who used a single toothbrush. Regression model indicated that boys were more likely to use multiple toothbrushes than girls (OR = 5.10, 95% CI 1.33to19.51, *p* < 0.05). Children whose toothbrushes were rated as heavy to extreme wear were approximately more likely to use multiple toothbrushes during a 3-month period (OR = 10.49, 95%CI3.21to34.26,*p* < 0.001). Children with limited social skills were less likely to use multiple toothbrushes than children with low social skills (OR = 0.25, 95% CI 0.09to0.74, *p* < 0.05, Table 4).

**Table 3:** Number of toothbrushes used by the recruited children over the past 3 months

No. of toothbrushes used in 3 months	No. of children (%)
1	252 (91.0)
2	16 (5.8)
3	3 (1.1)
4	3 (1.1)
5	2 (0.7)
6	1 (0.4)

**Table 4:** Characteristics of children who used multiple toothbrushes in 3 months

	Children used at least two toothbrushes in 3 months		
	OR (95% CI)	*p*-value	Multiple comparisons
Gender			
Male	5.10 (1.33, 19.51)	0.017	
Female^a^			
Diagnosis		0.032	(3), (4) > (2)
Autism (1)	0.32 (0.10, 1.05)	0.060	(1)=(2)
Developmental delay (2)	0.09 (0.01, 0.72)	0.024	(1)=(4)
DS, CP, or epilepsy (3)	1.32 (0.32, 5.43)	0.703	(1)=(3)
Multiple diagnoses (4)^a^			
Social skills		0.024	(3) > (2)
Average or high (1)	0.15 (0.02, 1.38)	0.093	(1)= (2)
Limited (2)	0.25 (0.09, 0.74)	0.012	(1)= (3)
Low (3)^a^			
Excessive toothbrush wear			
Yes	10.49 (3.21, 34.26)	< 0.001	
No ^a^			

Odds Ratio (OR) and 95% confidence interval (95% CI) were estimated by Binary Logistic Regression

^a^Reference group

### Overview of the collected toothbrushes

**Fig. 4:**
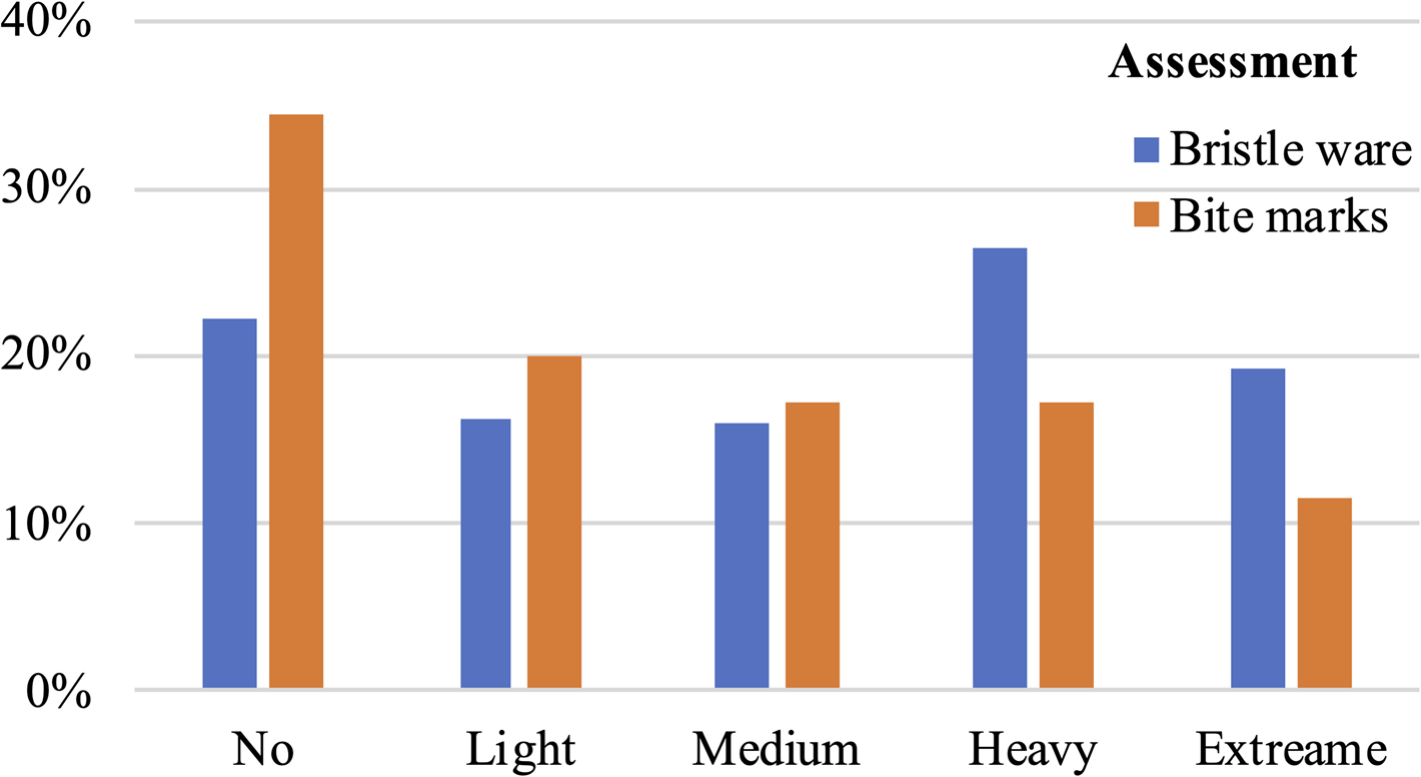
Toothbrush wear and bite marks on 321 toothbrushes used by children with special care needs

A total of 321 toothbrushes were collected. The wear and bite-mark score were 2.05 ± 1.45 and 1.52 ± 1.41, respectively. The wear score was positively correlated with bite-mark score (r = 0.68, *p* < 0.01). The wear score of toothbrushes with visible bite marks was significantly higher than that of toothbrushes without visible bite marks (2.67 ± 1.21vs.0.85 ± 1.05,*p* < 0.001). Distinct bite marks were present on 147(45.8%) toothbrushes, while blur marks were observed on 64(19.9%) toothbrushes (Fig. 4).

### Associations between toothbrush deterioration and participant characteristics

The toothbrushes used by 114(41.2%) children showed heavy to extreme wear. The presence of excessive wear was associated with children’s social skills and parents’ education background (*p* < 0.05). When comparing to children with average or high social skills, the toothbrushes used by children with limited social skills (OR = 2.69,95%CI1.11to6.53, *p* < 0.05) or low social skills (OR = 2.89,95%CI1.32to6.29, *p* < 0.01) were more likely to have excess wear. The toothbrushes used by children whose parents received 10–13 years’ education were less likely to show excess wear, when comparing to children whose parents’ highest educational attainments were no more than 9 years (OR = 2.80,95%CI0.15to0.79, *p* < 0.05). The presence of distinct bite marks was nearly two times likely to exist among children who showed challenging or resistant behaviors during toothbrushing (OR = 1.96,95% CI1.15to3.32, *p* < 0.05, Table 5).

**Table 5:** Factors associated with toothbrush deterioration

	Presence of heavy to extreme wear		Presence of distinct bite marks	
	OR (95% CI)	*p*-value	OR (95% CI)	*p*-value
Social skills		0.027		
Low	2.89 (1.32, 6.29)	0.008		
Limited	2.69 (1.11, 6.53)	0.028		
Average or high ^a^				
Toothbrushing is a difficult task				0.013
Yes			1.96 (1.15, 3.32)	
No ^a^				
Toothbrushing frequency				0.021
Twice daily or more			2.80 (1.31, 5.98)	0.008
Once daily			1.78 (0.81, 3.88)	0.150
Never/occasionally ^a^				
Parents’ education		0.040		
14 years or above	0.53 (0.26, 1.11)	0.532		
10–13 years	0.35 (0.15, 0.79)	0.012		
9 years or below ^a^				

OR and 95% CI were estimated by Binary Logistic Regression

^a^Reference group

### Parents’ comments about the toothbrushes used by children with special care needs

Seventy-five (27.1%) parents reported that the commercially available toothbrushes used by their children should be modified. Fourteen parents suggested that the texture, the length, the diameter, and the distribution of toothbrush bristles should be reasonably adjusted. The bristles should neither be too soft, too rough, too long, nor too stiff. Parents perceived that the multi-dimensional bristles could simplify the toothbrushing procedure (a single stroke was able to brush multiple tooth surfaces, if the toothbrush bristles covered on multiple surfaces). Several parents recommended that the angles of toothbrush handles should be modified so that children with special care needs could hold the toothbrush properly. Besides, the size of toothbrush heads should be more diverse so that parents could choose different toothbrushes to clean different tooth surfaces. Parents also suggested that the price of personally modified toothbrushes should be lower.

Parents expressed their concerns about children’s toothbrush-biting habit. They suggested that a soft rubber pad should be placed on the toothbrush head to protect those children who had toothbrush-biting habits. Besides, an ideal toothbrush should simplify the toothbrushing procedures, make it fun for children to brush teeth, and provide verbal or visual instructions to children while brushing teeth (Table 6).

**Table 6:** Parents’ suggestions to improve the design of toothbrushes used by their children

Items	Brief comments/suggestions
Bristles	• “The bristles are too long” • “The bristles are too thin” • “The bristles are too hard/stiff” • “The bristles are too soft, and the toothbrush cannot remove the debris efficiently” • “The bristles are too rough, and my child complains that toothbrushing causes pain” • “The bristles should be softer. The pressure of toothbrushing should not cause bleeding gums” • “The bristles should cover all the surfaces of the toothbrush head” • “Multi-dimensional bristles” • “Optimal bristle diameter” • “The procedure of toothbrushing might be simplified if there the bristles are distributed on the lateral sides of a toothbrush head”
Handle	• “The toothbrush handle is difficult for my child to hold” • “The angle of toothbrush handles should be adjusted, so that the back teeth could be reached easily”
Toothbrush head	• “Various sizes of the toothbrush head should be provided” • “The size of a toothbrush head should be adjusted properly, so that the back teeth could be brushed more efficiently. • “There should be a soft rubber pad on the toothbrush head. If children bite the toothbrushes, less damage will be caused to their teeth, and they can feel more comfortable”
Additional function	• “It is better if the toothbrush can make toothbrushing fun for kids (help my child love teeth brushing)” • “We need a toothbrush which can simplify the toothbrushing steps (brush more areas with a single stroke)” • “…show the toothbrushing steps or provide instructions to children by using verbal or visual hints” • “Toothbrushing instruction is needed”
Others	• “My child bites the toothbrushes, and the toothbrushes are distorted very soon” • “When I help my child to brush the buccal surfaces of the front teeth, my child does not know how to cooperate. I wish there is a toothbrush which is specialized in brushing the front teeth” • “The design of toothbrushes should make it more convenient for parents to help children brush their teeth” • “The individually-modified toothbrushes should be less expensive”

## Discussion

The deterioration of toothbrushes used by children with special care needs were investigated in this study. A total of 321 toothbrushes were collected from 277 children. Children used multiple toothbrushes in a 3-month period showed higher deterioration scores than children who used a single toothbrush. Over 40% children presented excessive bristle wear and distinct bite marks on their toothbrushes. The severity of toothbrush deterioration was associated with children’s social skills, toothbrushing behaviors, and parents’ education background. More than a quarter of the parents perceived that their children’s toothbrushes should be modified, and suggestions to improve the design of toothbrushes for children with special care needs were provided by parents. To the best of our knowledge, this was the first investigation to evaluate the toothbrush deterioration levels among preschool children with special care needs.

The extent of toothbrush wear was rated by a 5-point scale, which was proposed by Conforti et al., and further elaborated by Leeuwen et al. [9, 14] Leeuwen et al. collected 516 toothbrushes which had been used by adults, and they demonstrated that 38.8% of the collected toothbrushes had heavy to extreme wear [9]. Heavy to extreme wear were presented by 45.8% of the collected toothbrushes, indicating that excessive toothbrush wear was more likely to be observed among children with special care needs. Another popular index for the measurement of toothbrush wear was a 4-point scale validated by Rawls et al., which divided the toothbrushes into 4 categories: no signs of wear, low wear, medium wear, and high wear [13]. Garbin et al. reported that 19.5% of the toothbrushes used by preschool children exhibited medium wear, and 22.5% toothbrushes showed high wear [10]. In our study, 15.9% toothbrushes were rated as medium wear, 26.5% toothbrushes were rated as heavy wear, and 19.3% toothbrushes showed extreme wear. It was not feasible to make direct comparisons between the two different rating systems. However, the principal findings of those studies indicated that a portion of preschool children used severely worn toothbrushes. Their toothbrushes were not renewed, even though the tufts were splayed or matted. The awareness of timely toothbrush renewal should be raised among parents and other caregivers.

The excessive wear of toothbrush bristles was assumed to be associated with toothbrush-biting habit [9, 11].However, up to date, few studies have investigated the severity of toothbrush deterioration caused by toothbrush-biting. One significant contribution of this study was to introduce a validated 5-point scale to assess the distribution of bite marks on toothbrushes. By using the rating scale, the bite-mark scores and wear scores of the 321 toothbrushes were positively correlated (r = 0.68, *p* < 0.01). Additionally, 42.6% children showed distinct bite marks on their toothbrushes, and 65.7% of the collected toothbrushes exhibited visible bite marks. The wear score of toothbrushes with visible bite marks was significantly higher than that of toothbrushes without visible bite marks. Those findings supported the assertion that chewing or biting toothbrushes whilst brushing could speed up the process of toothbrush wear. Those findings also revealed that toothbrush-chewing or toothbrush-biting was a common phenomenon among young children with special care needs.

Another contribution was to explore the factors associated with the deterioration of children’s toothbrushes. The main findings illustrated by the regression model indicated that the type of impairment presented by the recruited children was associated with the number of toothbrushes used in a 3-month period. Children with developmental delay were more likely to use a single toothbrush, while children who were diagnosed with cerebral palsy, Down syndrome, epilepsy, or multiple diagnoses tended to use 2 to 6 toothbrushes in a 3-month period. This was consistent with the previous findings. Ortega et al. reported that children with cerebral palsy were likely to have the habit of biting objects, and the toothbrushes were vulnerable to be deteriorated, as those children might bite the toothbrushes during brushing [11, 12].

Over half of the parents reported that toothbrushing was a difficult task for their children. The regression analysis suggested that distinct bite marks were nearly two times likely to be presented on the toothbrushes that had been used by children who showed challenging or resistant behaviors during tooth brushing. Besides, when comparing to children with high or average social skills, excessive toothbrush wear was more likely to be observed among children with low social skills (OR = 2.89, 95%CI 1.32to6.29), or children with limited social skills (OR = 2.69, 95% CI 1.11to6.53). Social skills refer to the “interpersonal skills, social responsibility, self-esteem, gullibility, naïveté (i.e., wariness), social problem solving, and the ability to follow rules/obey laws and to avoid being victimized” [16]. Children with sub-average social skills were less likely to follow the instructions given by the caregiver, and they were likely to brush their teeth with improper toothbrushing techniques. If children brushed their teeth with uneven strokes, their toothbrush bristles could be matted or splayed quickly [11].The prior study also revealed that children with low social skills showed poorer toothbrushing performance than their peers, and toothbrushing trainings in early childhood were recommended for children with special care needs [17, 18]. The design of toothbrushes could also influence the deterioration levels of toothbrushes [4, 19]. Parents’ comments reflected that the design of the toothbrushes used by children with special care needs should be improved, for instance, the bristles should not be too long, too stiff, nor too soft. The angle of the handles, and the size of the toothbrush heads should be adjusted, making it more convenient and more comfortable to brush the back teeth. The additional function was also recommended by parents, including putting a soft rubber pad to protect children who had toothbrush-biting habits, providing verbal or visual instructions to children whilst brushing teeth, and adding multi-dimensional bristles to simplify the toothbrushing procedures.

The sampling method was the main limitation of this study. A convenience sample of 277 children with special care needs was recruited. Children whose parents signed the consent forms and collected the used toothbrushes were all included. We did not screen the participants based on their background information. Therefore, the sex ratio was not matched in this study (67.5% were boys). The number of children who were diagnosed with Down syndrome, cerebral palsy, or epilepsy was smaller than children who were diagnosed with autism or developmental delay. Another limitation of this study was that the recruited children were mainly diagnosed with neurological conditions, for instance, Down syndrome, cerebral palsy, epilepsy, autism or developmental delay. Children with systemic disorders, affective disorders and/or severely physical handicap were not included. In further investigations, more case-control studies are warranted to compare the toothbrush deterioration among children with various developmental profiles.

## Conclusions

The principal findings suggested children with special care needs were likely to bite their toothbrushes. The presence of excessive deterioration was associated with children’s social skills, toothbrushing behaviors, and parents’ educational attainment. The awareness of timely toothbrush replacement for children with special care needs should be raised among parents or caregivers. The commercially available toothbrushes should be modified to meet the additional needs of young children.

## Supplementary information

**Additional file 1.** STROBE Statement—checklist of items that should be included in reports of observational studies.

## Data Availability

The datasets are available from the corresponding author upon reasonable request.

## Abbreviations

SCCC: Special Child Care Center
OR: Odds ratio
CI: Confidence interval
